# Genipin Delays Corneal Stromal Enzymatic Digestion

**DOI:** 10.1167/tvst.10.9.25

**Published:** 2021-08-23

**Authors:** Christopher Donovan, Elena Koudouna, Curtis E. Margo, Marcel Y. Avila, Edgar M. Espana

**Affiliations:** 1 Department of Ophthalmology, School of Medicine, Universidad Nacional de Colombia, Hospital Universitario Nacional, Bogota, Colombia; 2 Structural Biophysics Group, School of Optometry and Vision Sciences, Cardiff University, Cardiff, UK; 3 Department of Pathology and Cell Biology, Morsani College of Medicine, University of South Florida, Tampa, FL, USA; 4 Department of Ophthalmology, Morsani College of Medicine, University of South Florida, Tampa, FL, USA; 5 Molecular Pharmacology and Physiology, Morsani College of Medicine, University of South Florida, Tampa, FL, USA

**Keywords:** collagenase, stroma, fibroblasts, wound, genipin

## Abstract

**Purpose:**

To evaluate the use of genipin in delaying enzymatic digestion of corneal stroma.

**Methods:**

Human corneal stromal tissue was treated with genipin, a known chemical crosslinker, and then along with control tissue was subjected to enzymatic digestion with collagenase. The effects of genipin treatment in retarding stromal digestion were analyzed with phase contrast microscopy, a protein quantification assay, second harmonic generation imaging, and transmission electron microscopy.

**Results:**

Genipin increased stromal resistance to enzymatic digestion when compared with untreated stroma. A morphologic analysis and protein quantification showed increased stromal resistance to enzymatic digestion once stromal tissue was treated with genipin. Second harmonic generation imaging revealed persistent fibrillar collagen signaling in genipin-treated tissue in contrast with untreated tissue suggesting that genipin retards collagenolysis.

**Conclusions:**

Genipin increases stromal resistance to enzymatic digestion in controlled experiments as demonstrated by protein quantification studies and through morphologic imaging.

**Translational Relevance:**

This study explores the novel use of genipin in delaying enzymatic stromal digestion. Delaying stromal melting in the setting of corneal infectious or autoimmune keratitis can potentially decrease clinical morbidity.

## Introduction

Progressive corneal ulceration is a sight-threatening condition that can lead to corneal perforation and permanent loss of vision. Multiple disorders including viral, bacterial, and fungal infections, as well as chemical injuries and autoimmune disease, can result in severe destruction of stromal tissue.

The aim of treatment in persistent or progressive corneal ulceration is to stop the loss of stromal tissue, promote epithelialization, and avoid corneal perforation. The management of progressive stromal damage is largely limited to the treatment of the underlying infectious organism or inflammatory condition. Vitamin C supplementation and low-dose tetracycline antibiotics are commonly used to prevent or slow corneal ulceration, but evidence that these therapies are effective is sparse.^1^^–^^6^ Corneal transplantation is required when corneal perforation is imminent or has occurred.

Progressive corneal ulceration during infectious keratitis is a complex and multifactorial process where both infecting organisms and the host's immune system play roles in matrix destruction. The eradication of bacteria by antimicrobials does not guarantee the end of stromal destruction or prevent perforation. The most common bacteria associated with corneal ulcers in contact lens users, *Pseudomonas aeruginosa*, inflicts injury via different proteases, for example, *Pseudomonas aeruginosa* small protease.^7^^,^^8^ Similar proteases have been found in other infectious pathogens that frequently cause corneal ulcers such as *Staphylococcus aureus*, *Fusarium*, *Aspergillus*, and *Candida*.^9^^–^^15^

In addition to collagenases produced by infectious organisms, the host immune system also secretes enzymes, cytokines, and reactive oxygen species as part of the immune response to infection. A similar list of culpable proteins are responsible of stromal destruction in autoimmune keratitis. These host-derived collagenases produced by corneal keratocytes and epithelial cells as well as infiltrating neutrophils are important factors in wound repair following injury.^4^^,^^16^^–^^21^

Genipin is a crosslinking compound isolated from *Gardenis jasminoides* and *Genipa americana*, also known as the gardenia and genipap fruits.^22^^–^^25^ Previous studies have demonstrated that topical genipin solutions can be safely used to crosslink corneal stromal tissue with minimal risk of toxicity.^24^^,^^26^^,^^27^ In this project, we investigate genipin as a potential corneal stromal stabilizing agent in the presence of bacterial obtained collagenase.

## Methods

### Stromal Discs Dissolution in Collagenase Type 1

**Figure 1.**
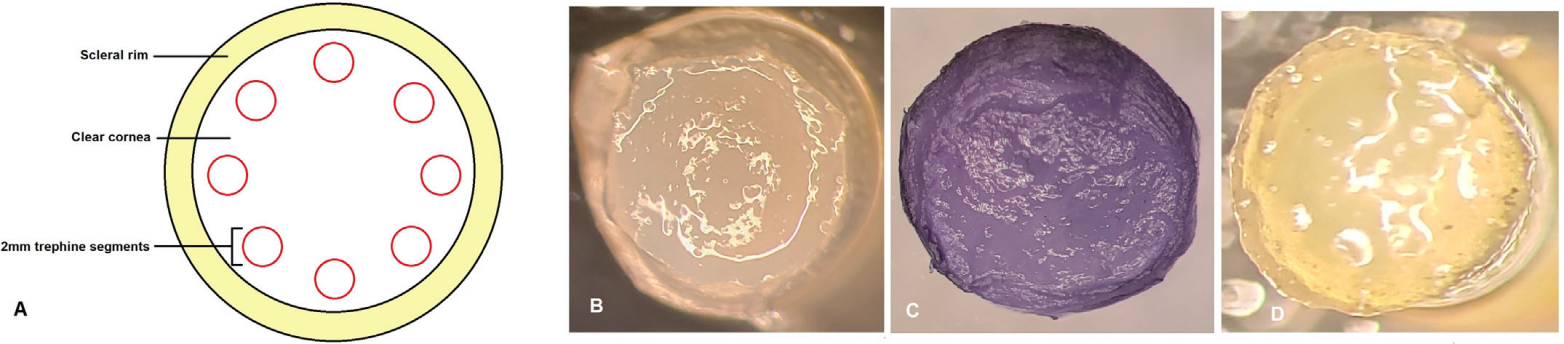
Stromal discs were obtained from human corneas and treated with genipin. (A) Diagram illustrates technique used to obtain 2-mm stromal discs/fragments. (B) A 2-mm stromal disc punched out from donor cornea. (C) Stromal disc acquires purple coloration after exposure to genipin for 1 hour with no noticeable change in diameter. (D) Purple coloration fades away when the stromal disc is place in a clear balanced salt solution.

**Figure 2.**
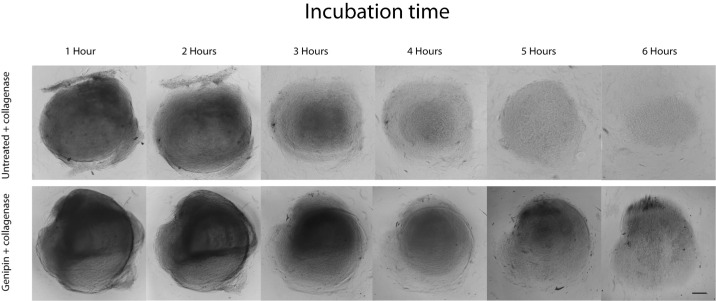
Phase contrast photographs show increased stromal resistance to enzymatic digestion after genipin treatment. Stromal disc enzymatic digestion without genipin treatment (Top images) and after genipin treatment (bottom images) at different hour intervals. Stromal discs used in this image were obtained from a 47-year-old donor cornea. Bar = 350, µm.

Seven human corneas from donors aged 32 to 72 years old and maintained at 4 °C in Optisol (Chiron Vision, Irvine, CA) for less than seven days after death were obtained from the Tampa Lions Eye Bank (Tampa, FL) and handled according to the tenets of the Declaration of Helsinki. The epithelium of each cornea was removed with a 15° blade. Eight 2-mm diameter stromal discs were trephined from each cornea using a 2 mm disposable dermatology punch (Integra Miltex, York, PA) (Fig. 1). From each cornea, four trephined stromal discs were immersed for 1 hour in 0.25% genipin (Sigma Aldrich, St. Louis, MO) dissolved in dimethyl sulfoxide (DMSO, Sigma Aldrich) at room temperature. The remaining four trephined stromal discs were immersed in a control solution containing only DMSO at room temperature. All stromal discs were then subsequently washed for 1 hour in DMSO. All stromal discs were then immersed in a 1% collagenase type 1 from *Clostridium histolyticum* in Dulbecco's modified eagle media (DMEM) solution and kept on a rotating incubator set at 37 °C. During the enzymatic reaction process, stromal discs were observed hourly for the first hours. Photographs were taken of both genipin-treated and untreated discs using a phase contrast microscope (Leica DM5500B) (Fig. 2). Identical conditions were used to facilitate comparisons between samples.

### Total Protein Assay During Stromal Disc Dissolution in Collagenase

Stromal discs from three donor corneas, aged 42, 64 and, 67 years, were prepared and treated as described elsewhere in this article. Genipin-treated and untreated stromal discs were placed in a 96-well dish and allowed to dissolve at 37 °C in 0.1% collagenase dissolved in TESCA buffer (50 mM TES buffer with 0.36 mM calcium chloride, pH 7.4), according to manufacturer instructions (Sigma Aldrich). We used 0.1% collagenase solution in TESCA buffer to limit background interference in the protein assay. We took 25 µL aliquots of collagenase solution at hours 1, 3, 5, and 6 from the genipin-treated and untreated groups. The total protein content of these aliquots was quantified using a BCA total protein assay catalog number BCA1 (Sigma Aldrich), using a plate reader.

### Second Harmonic Generation Microscopy of Digested Stromal Discs

Stromal discs were trephined from two donor corneas, ages 26 and 38 years. Two unprocessed stromal discs from each cornea were not exposed to genipin or DMSO and were imaged as controls. The remaining stromal discs from each cornea were prepared as described elsewhere in this article. Two genipin-treated and two untreated discs from each cornea were immersed in 1% collagenase solution at 37 °C for 2 hours before imaging. Two genipin-treated discs from each cornea were imaged without digestion in collagenase. Flat mounts of each disc, including normal untreated discs, were imaged using an Olympus MPE-RS microscope using a 25× (0.95 NA) water-immersion objective (Olympus). Propidium iodine (Thermo Fisher Scientific, Waltham, MA) in a 1:100 concentration was added to the Optisol solution at the time of imaging. Two-photon second harmonic generation (SHG) signals were generated using a mode-locked titanium:sapphire laser at 960 nm. The SHG forward-scattered signals passing through the corneal sections were collected using a 0.8 NA condenser lens with a narrow band-pass filter (465–485 nm). Backward-scattered SHG signals were detected with a band pass filter (460–500 nm). All samples were scanned using a 2-µm *z*-axis step size from the back to the front of the section. The two-photon excited fluorescent signal from propidium iodine was captured with a band pass filter (575–630 nm).

### Transmission Electron Microscopy (TEM)

Microphotographs obtained using TEM from three donor corneas, ages 37, 40, and 45 years, were analyzed for morphologic study. Stromal discs from each cornea were prepared as described elsewhere in this article. Two genipin-treated and two untreated discs from each cornea were immersed in 1% collagenase solution at 37 °C for 2 hours before processing for TEM. Two genipin-treated and two untreated discs from each cornea were left in DMEM without collagenase for 2 hours. Stromal discs were fixed in 4% paraformaldehyde, 2.5% glutaraldehyde, 0.1 M sodium cacodylate, pH 7.4, with 8.0 mM CaCl_2,_ post-fixed in 1% osmium tetroxide. The discs were dehydrated in graded ethanol series, followed by propylene oxide. The tissue samples were infiltrated and embedded in a mixture of Embed 812, nadic methyl anhydride, dodecenyl succinic anhydride, and DMP-30 (Electron Microscopy Sciences, Hatfield, PA). Thin sections (∼80 nm) were cut with a Leica ultramicrotome and poststained with 2% aqueous uranyl acetate and 1% phosphotungstic acid, pH 3.2. The sections were examined at 80 kV with a JEOL 1400 transmission electron microscope equipped with a Gatan Ultrascan US1000 2K digital camera.

### Fibril Area and Fibril Density

There were 100,000× TEM digital images from each stromal disc that were masked randomly. A region of interest of appropriate size was chosen within each image where fibrils were perpendicular/cross-sectional to the viewing plane. Cross-sectional fibril area, minimum cross-sectional fibril diameter, and total fibril density within each region of interest were measured using ImageJ software (National Institutes of Health, Bethesda, MD). The fibril density is listed as the percentage of each region of interest occupied by collagen fibrils.

### Statistical Analysis

Microsoft Excel 2013 (Redmond, WA) and Graph Pad Prism (San Diego, CA) were used for data analysis and to create histograms of minimum cross-sectional fibril diameter and cross-sectional fibril area from TEM images. The Student *t* test was used to draw statistical inferences when comparing the mean of continuous dependent variables. A *P* value of less than 0.05 was considered statistically significant.

## Results

### Genipin Treatment Retards Stromal Dissolution in 1% Collagenase

When both genipin-treated and untreated 2-mm stromal discs were digested in 1% collagenase solution in DMEM at 37 °C, stromal discs not treated with genipin consistently dissolved after 4.1 hours based on phase contrast microscopy. Stromal discs previously treated with genipin required an average of 7 hours to fully dissolve (*P* < 0.01). Microscope photographs obtained hourly during the period of observation showed progressive stromal disorganization and dissolution that progressed more rapidly in corneas not treated with genipin (see Fig. 2).

We noted that in all seven corneas, and regardless of the age of the donor tissue, the genipin-treated tissue was more resistant to digestion. Supplementary Figure S1 shows another example from a 40-year-old donor.

### Genipin Treatment Slows Down Stromal Proteolysis in 0.1% Collagenase

**Figure 3.**
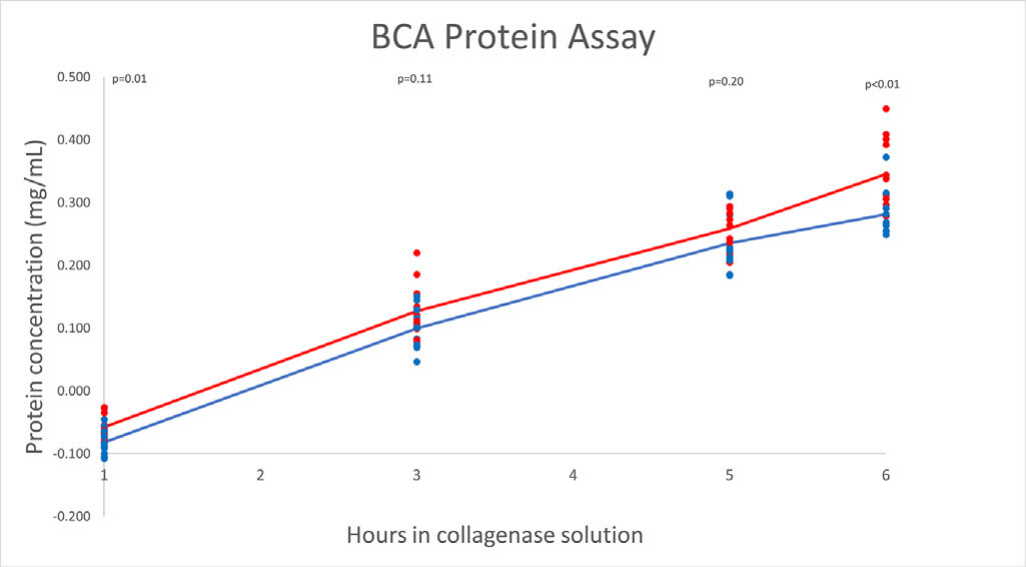
Genipin decreases proteolysis and enzymatic stromal dissolution. Protein concentration in milligrams er milliliter from 1 through 6 hours obtained during immersion in 0.1% collagenase solution measured by BCA protein assay. The trendline for genipin-treated discs shows decreased protein concentration over the first 6 hours of digestion compared with untreated discs. Corneas were obtained from three different donors. The *blue* *line* corresponds with genipin-treated tissue before collagenase digestion. The *red* *line* corresponds with collagenase digestion only without genipin pretreatment.

To compare the differences in stromal proteolysis after genipin treatment, we measured protein concentration in the 0.1% collagenase solution where the stromal discs were digesting using a BCA assay kit, with the assumption that the protein levels in the solution will increase with progressive disc dissolution. Genipin-treated stromal discs showed decreased protein concentration over the first 6 hours compared with stromal discs not treated with genipin (see Fig. 3). These results correspond with observational data showing increased time to total dissolution in genipin-treated corneas, namely, that the protein content in the solution where the stromal discs were digested steadily increases with stromal protein digestion. Among individual donors, slightly increased protein concentrations were found in discs obtained from the 42-year-old donor compared with two donors aged 64 and 67 years. Although this increased protein concentration was detected in both genipin-treated and untreated discs, a higher protein concentration was revealed in untreated discs relative to genipin-treated discs. Supplementary Figure S2 shows each tissue independently to demonstrate the effect of genipin despite tissue age.

### Stromal Hierarchical Organization and Fibrillar Damage

**Figure 4.**
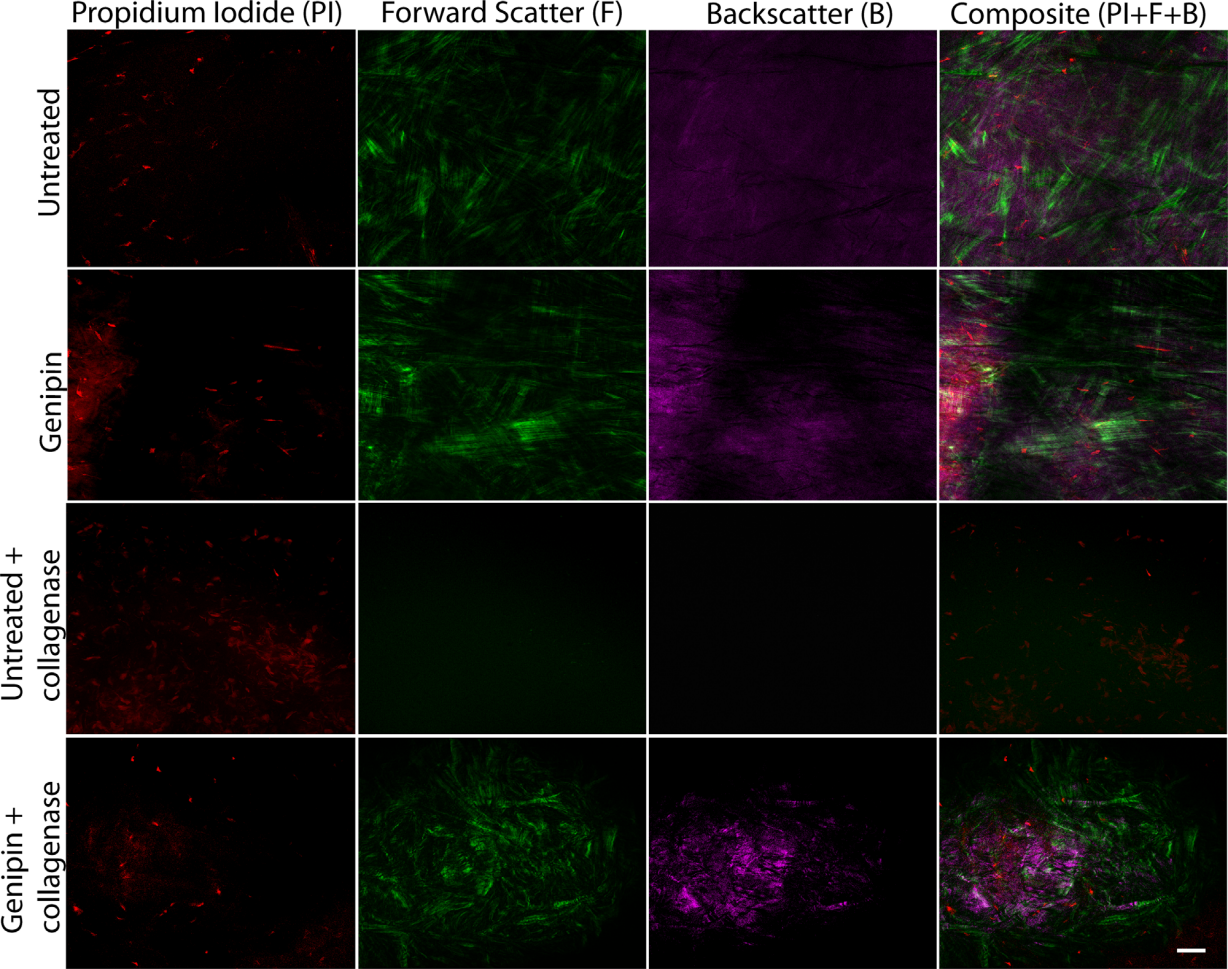
Genipin delays stromal matrix enzymatic digestion and collagenolysis, flat-mount evaluation in anterior–posterior axis. Images obtained after genipin only treatment show normal keratocyte cell nuclei, preservation of lamellae with normal forward-scatter and back-scatter signal compared with unprocessed corneal tissue (*first* *and second rows*). After immersion in 1% collagenase solution for 2 hours, untreated stromal tissue shows severe damage to the stromal architecture and collagenolysis with loss of forward-scatter and back-scatter signaling suggestive of collagen fibril damage (*third* *row*). Tissue treated with genipin before immersion in collagenase solution shows the effects of genipin in delaying stromal damage with enzymatic digestion. Forward-scatter and back-scatter signaling suggest preservation of collagen fibrils (*fourth* *row*). Stromal discs used for this image were obtained from a 26-year-old donor cornea. Bar = 50 µm.

To assess stromal structure and hierarchical organization after genipin and collagenase digestion, we performed SHG imaging with forward-scatter and back-scatter settings in flat mounts samples. In representative captured images, normal control corneas showed flat keratocyte nuclei that were embedded between lamellae and a distinct pattern of well-organized structures like lamellae were evident with forward SHG signal. Back-scatter SHG signals seemed to be mild and diffuse, without any localized area of increased signaling. A group treated only with genipin showed no significant changes compared with normal tissue. Lamellar organization was well preserved, as shown by forward signaling. It was noted that keratocytes were embedded between the matrix. After 2 hours of immersion in collagenase solution, the untreated group showed severe anatomic disruption with marked loss of forward- and back-scatter signals, a finding consistent with loss of stromal structure due to collagenolysis. Genipin-treated stroma better resisted enzymatic digestion as demonstrated by preservation of stromal anatomy as shown by SHG signaling, a clear indicator that fibrillar collagen was preserved after genipin treatment (see Fig. 4). Taken together, these data show a correlation between the severity of stromal damage and fibrillar collagen signal loss after collagenase digestion in stromal tissue. Two hours after collagenase digestion, stromal structure is better preserved in genipin-treated tissue than control tissue.

### Electron Microscopy

**Figure 5.**
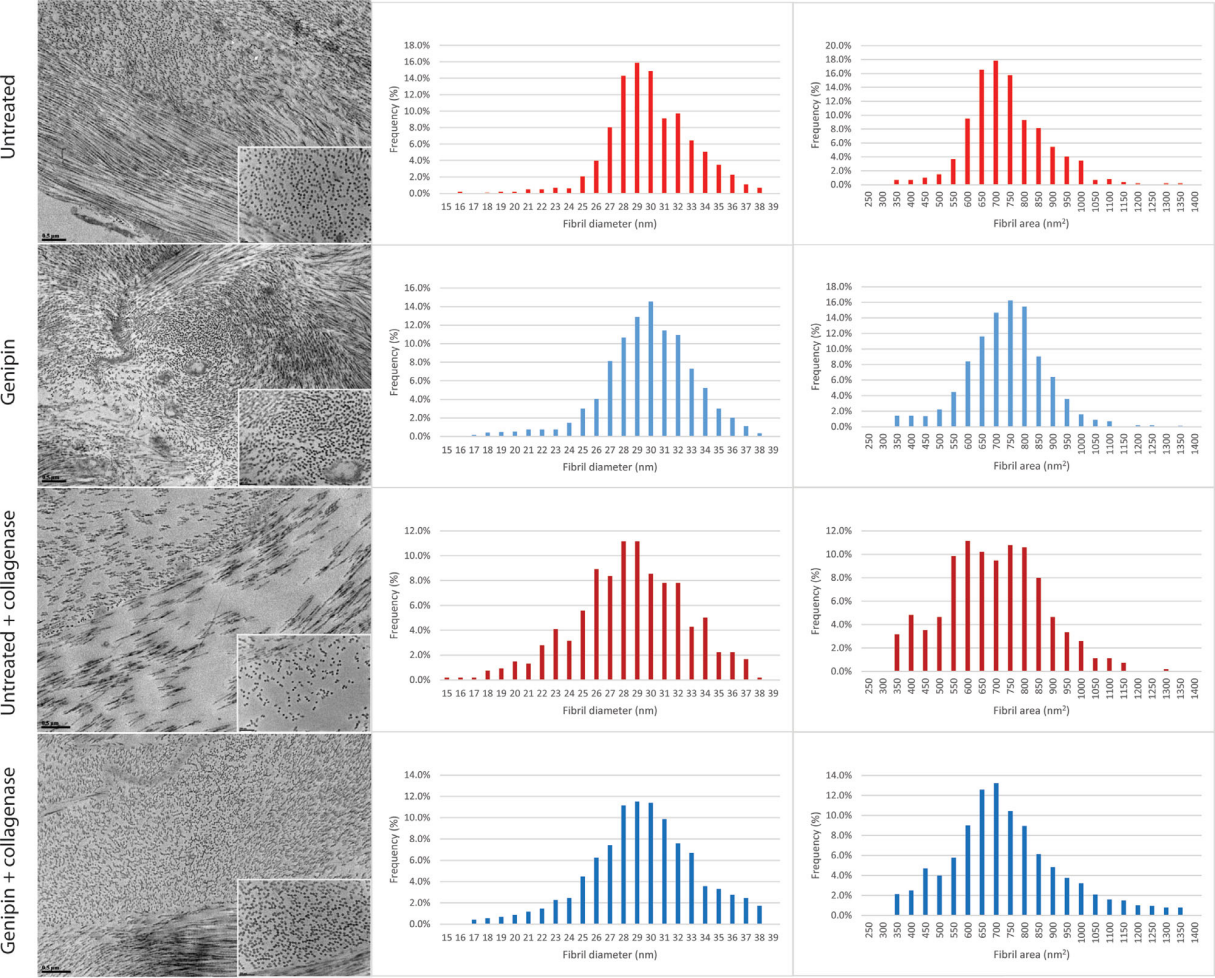
Two microphotographs—30,000× (larger image) and 100,000× (smaller inlay image)—from an electron microscope of untreated and genipin-treated stromal discs. After collagenase digestion, untreated tissue shows a decreased number of collagen fibrils, decreased length of collagen fibrils, and increased heterogeneity in diameter. Genipin treatment before digestion in collagenase solution demonstrates greater preservation of fibril and stromal anatomy as well as organization. Histograms emphasize a shift toward decreased minimum fibril diameter and fibril area after two hours immersion in collagenase solution which is more pronounced in untreated stromal discs. Corneal stromal discs for this ultrastructural study were obtained from a 40-year-old donor. Bar = 0.5 µm for 30,000× images and 200 nm for 100,000× images.

Two hours after dissolution in collagenase solution, untreated stromal disc microphotographs showed substantial loss of collagen fibrils, decreased fibril diameter, and variability in fibril length. In contrast, microphotographs of stromal discs treated with genipin displayed significantly decreased degrees of collagen fiber loss and more preserved fibril diameter and fibril length (Fig. 5). Each of these findings suggest that genipin treatment preserves the corneal collagen structure and content after exposure to bacterial collagenase.

### Fibril Area and Fibril Density

Before dissolution in collagenase solution, the cross-sectional area and minimum cross-sectional diameter of individual collagen fibrils were both slightly smaller in stromal discs treated with genipin compared with untreated discs. Fibril density was slightly higher in stromal discs treated with genipin compared with untreated discs; however, none of these differences were statistically significant. After 2 hours of immersion in collagenase solution, cross-sectional fibril area, minimum cross-sectional fibril diameter, and fibril density decreased slightly in genipin-treated tissue. In contrast, a substantial and statically significant decrease was found in the cross-sectional area, minimum cross-sectional diameter, and fibril density of untreated tissue after 2 hours in collagenase solution compared with untreated tissue before dissolution. Significant decreases in each measurement were also found in untreated stromal discs after 2 hours in collagenase solution compared with genipin-treated stromal discs after two hours in collagenase solution (Table). Histograms highlight a shift toward decreased minimum fibril diameter and fibril area after 2 hours of immersion in collagenase solution which is more pronounced in untreated stromal discs compared with genipin-treated discs (Fig. 5).

**Table.** Average Cross-Sectional Size of Individual Collagen Fibrils and Fibril Density of Each Region of Interest Occupied by Collagen Fibrils. After 2 Hours of Immersion in Collagenase Solution Untreated Tissue Displayed Greater Decreases Both in Average Cross-Sectional Fibril Size and Fibril Density Compared With Tissue Treated With Genipin.

	Minimum Fibril Diameter (nm)		Fibril Area (nm^2^)		Fibril Density (%)	
	Before Collagenase	After Collagenase	Before Collagenase	After Collagenase	Before Collagenase	After Collagenase
Untreated	29.42	28.00 (*P* < 0.01)	712.54	667.39 (*P* < 0.01)	24.62	18.45 (*P* = 0.02)
Genipin	29.26	28.88 (*P* < 0.01)	709.97	709.54 (*P* = 0.94)	25.85	24.90 (*P* = 0.47)
	(*P* = 0.33)	(*P* < 0.01)	(*P* = 0.64)	(*P* < 0.01)	(*P* = 0.39)	(*P* = 0.01)

## Discussion

Therapies that retard progressive stromal dissolution will decrease ocular and visual morbidity and decrease the need for corneal transplantation. Such therapies should be a research priority. Different therapies used to limit stromal ulceration and scar formation (e.g., topical steroids, vitamin C, and oral doxycycline) have not demonstrated consistent beneficial outcomes.^28^^–^^31^ Oral vitamin C and doxycycline have been shown to have some benefit in treating corneal melt; however, robust clinical evidence is lacking and oral doxycycline can have side effects.^1^^–^^3^^,^^6^^,^^32^ Other adjuvant therapies, such as chelating agents, enzyme inhibitors, and other anti-inflammatory agents remain investigational.^33^^–^^42^

Genipin in rabbit corneas increases stiffness and resistance to stress compared with control tissues.^43^ Topical application of genipin has resulted in corneal flattening ex vivo.^43^ These findings have been replicated in porcine corneas. Ocular rigidity measurements have also shown similar results in eyes treated with or without an intact epithelium.^44^^,^^45^ Porcine corneas crosslinked by genipin have been found to have significantly increased resistance to digestion by bacterial collagenase compared with controls.^45^^,^^46^ Compared with UV-induced crosslinking, topical genipin has shown less endothelial toxicity and less reductions in corneal nerve and keratocyte densities.^24^^,^^26^^,^^27^ Following subtenon's injections of genipin, no cytotoxic effects have been found in scleral, choroidal, or retinal cells.^47^

Based on previous work on genipin demonstrating its efficacy as matrix crosslinker and its safety in different mammal tissues, we have continued to investigate the use of genipin for increasing corneal resistance to collagenase stromal digestion. We believe that retarding collagenase digestion has significant translational potential and that genipin could be used to slow corneal melting associated with infectious and autoimmune corneal ulceration. Several hypotheses on how genipin may retard stromal collagenolysis have been proposed: (1) increased host tissue resistance to digestion; (2) decreased microbial pathogenicity by altering microbe enzymatic efficacy; (3) increased host stromal extracellular matrix synthesis; and (4) interaction and potentiation of the action of antimicrobials.

Our study shows that one of the main mechanisms by which genipin works is through a decrease in the enzymatic digestion of healthy stroma. We have shown that treatment with genipin increases the resistance of host stroma to digestion by bacterial collagenase, thereby decreasing the enzymatic degradation of uncompromised healthy stromal matrix. Increased resistance of stromal tissue to enzymatic degradation could prevent more extensive damage to the eye, including extension to sclera or to intraocular tissues. It is noteworthy that collagenase type I used in this study may contain caseinase, clostripain, and tryptic activities. Genipin could, therefore, be effective against the enzymatic action of other proteases.

Interestingly, genipin crosslinking has been shown to modulate gene and protein expression as well. This finding may be significant in the pathogenesis of stromal ulceration, where the upregulation of matrix synthesis might occur in response to tissue destruction. Decreased expression of key *H* *pylori* virulence factors encoded by vacA and cagA genes was observed in the same mouse model along with decreased production of host-derived IFN-γ, IL-1β, and IL-8.^48^ Genipin has also been shown to decrease invasive potential and replication of RNA viruses in rotavirus models in the gastrointestinal tract.^49^

Crosslinking via UV light coupled with riboflavin or other photosensitizers has been used successfully as an adjuvant for treatment of corneal ulcers.^50^^–^^54^ The use of rose Bengal photodynamic antimicrobial therapy has shown effectiveness not only against bacterial pathogens but also in fungal and amoebic keratitis.^53^ However, we believe that chemical crosslinkers such as genipin have additional potential advantages over UV light-guided crosslinking methods. Although UV light-guided methods have proven effective in treating infectious ulceration, special equipment and the use of a sensitizer is required. Another advantage of genipin is that the application of UV light to severely thinned corneas poses a risk to corneal endothelial cells and intraocular tissue and can preclude its use.^50^^–^^52^^,^^55^^–^^57^

This study is the first to explore the use of genipin in an ex vivo model with human corneal tissue. It encourages further investigation into its use as a topical chemical crosslinker in the management of several diseases that cause corneal ulceration and melting. Several questions still need to be addressed, including how to optimize the timing and application of genipin. In addition, there was some variability in the results between corneas attained from donors of different ages. Although examining age-dependent variation was beyond the scope of this study, corneas obtained from younger donors were found to have increased protein concentration during dissolution in collagenase solution as measured by BCA assay. This increase was found to be slightly more pronounced in untreated stromal discs compared with genipin-treated stromal discs. Additional controlled experiments using age-stratified tissues are needed to further elucidate the effect that age has on the efficacy of chemical crosslinking of corneal tissue with genipin. Overall, the results of this study indicate that the reinforcement of corneal tissue through the use of chemical crosslinkers such as genipin could be a viable means of reducing corneal morbidity and the risk of perforation and scarring in persons with a variety of keratopathies.

## Supplementary Material

Supplement 1

Supplement 2
