# Supplementary material for: Genipin Delays Corneal Stromal Enzymatic Digestion
Source: Transl Vis Sci Technol. 2021 Aug 23;10(9):25. doi: 10.1167/tvst.10.9.25 (PMC8394563; doi:10.1167/tvst.10.9.25)
Supplement: Supplement 1 [file tvst-10-9-25_s001.pdf]

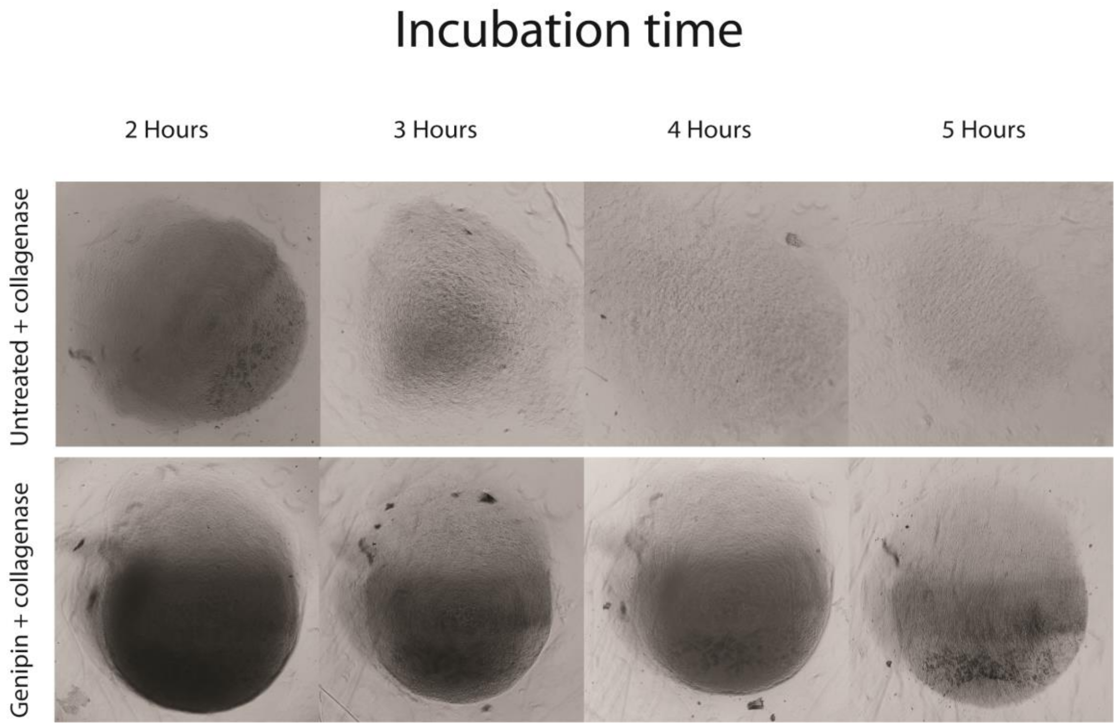

Supplementary Figure 2. Another example demonstrating that genipin retards collagenase digestion in donors of different ages. This tissue obtained from a 40-year-old donor was divided into different discs. Genipin treatment prior to digestion in collagenase solution demonstrates greater preservation of tissue.
