# Supplementary material for: Genipin Delays Corneal Stromal Enzymatic Digestion
Source: Transl Vis Sci Technol. 2021 Aug 23;10(9):25. doi: 10.1167/tvst.10.9.25 (PMC8394563; doi:10.1167/tvst.10.9.25)
Supplement: Supplement 2 [file tvst-10-9-25_s002.pdf]

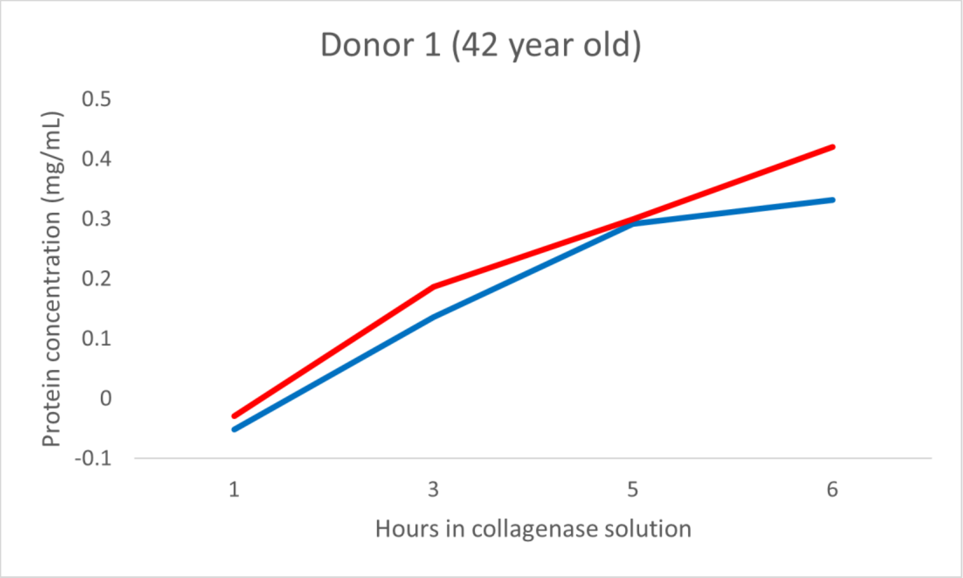

Donor 1 (42 year old)
Protein concentration (mg/mL)
0.5
0.4
0.3
0.2
0.1
0
-0.1
1
3
5
6
Hours in collagenase solution

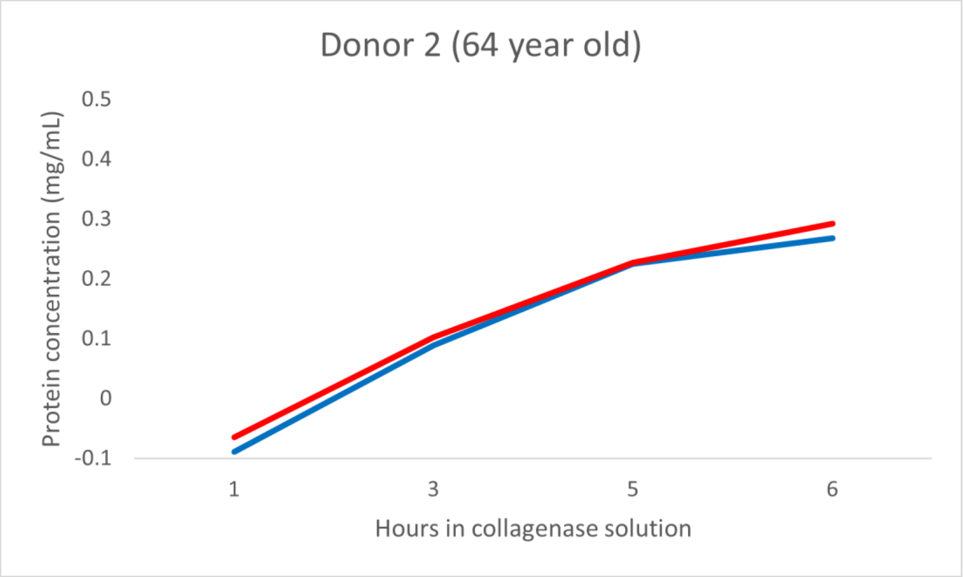

Donor 2 (64 year old)
Protein concentration (mg/mL)
0.5
0.4
0.3
0.2
0.1
0
-0.1
1
3
5
6
Hours in collagenase solution

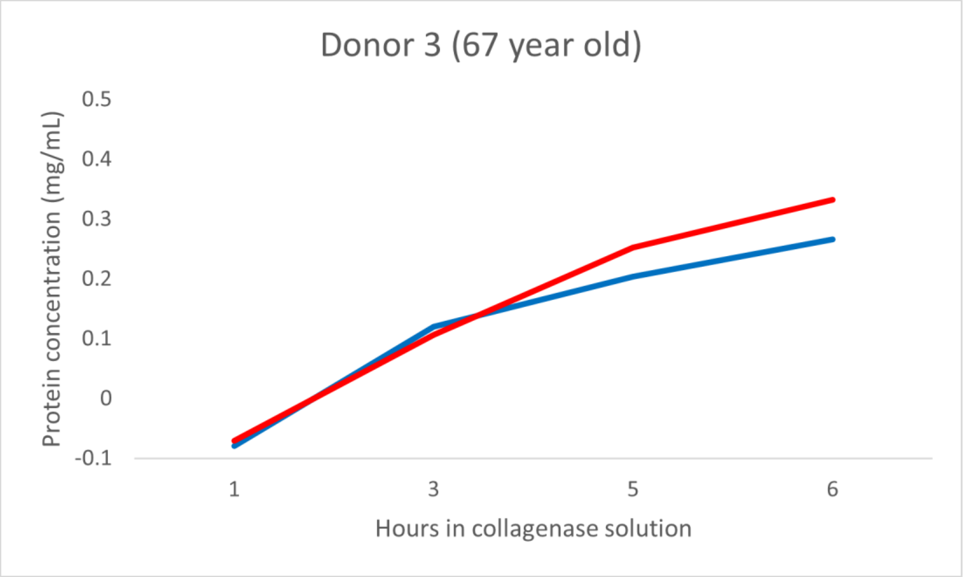

Supplementary Figures 3A, 3B and 3C. Genipin retards collagenase digestion of stromal tissue no matter age of tissue donor. Protein concentration in mg/ml from 1 through 6 hours obtained during immersion in 0.1% collagenase solution measured by BCA Protein Assay was obtained in three different donors.
